# Association between a pyroelectric infrared sensor monitoring system and a 3-dimensional accelerometer to assess movement in preweaning dairy calves

**DOI:** 10.3168/jdsc.2023-0393

**Published:** 2023-10-06

**Authors:** N. Sonntag, S. Borchardt, W. Heuwieser, F. Sutter

**Affiliations:** Clinic for Animal Reproduction, Faculty of Veterinary Medicine, Freie Universität Berlin, 14163 Berlin, Germany

## Abstract

The objective of this study was to correlate movement assessed by a pyroelectric infrared sensor system in preweaning dairy calves with lying and standing time assessed by a 3D accelerometer considering the temperature-humidity index (THI). A total of 35 dairy calves (1–7 d of age) were enrolled in the study and 20 calves were included in the final analyses. The lying and standing time of the calves was monitored with a 3D accelerometer (Hobo Pendant G Data Logger, Onset Computer Corporation, USA), which was used as the gold standard reference. The infrared sensor monitoring system (IMS; Calf Monitoring System, Futuro Farming GmbH, Germany) was fixed to the fence of the calf hutch within the calf's reach. Temperature-humidity was monitored with 2 validated THI sensors inside and on outside of each calf hutch. Additionally, one THI sensor was located near the calf hutches. The observation period lasted 14 consecutive days. The average standing time assessed by the 3D accelerometer was 13.4 ± 12.7 (mean ± standard deviation) min/h and the average lying time was 46.6 (±12.7) min/h. The median (25th percentile; 75th percentile) number of movements measured by the IMS was 360 (60; 919) movements per hour. Number of movements per hour measured by the IMS was compared with data obtained with a validated 3D accelerometer. The Pearson correlation coefficient between both standing and lying time and the number of movements was r = 0.85 and r = −0.85, respectively. The Pearson correlation coefficients were only slightly influenced by THI (low THI [<68]: r = 0.86; medium THI [68–72]: r = 0.85; high THI [>72]: r = 0.81). Our data show that the number of movements of dairy calves measured by IMS were highly correlated with the chosen gold standard reference method. High THI slightly affects the measurement accuracy of IMS.

## Highlights

• An IMS assessed movement in preweaning dairy calves.
• The movements of dairy calves measured by the IMS were highly correlated with the reference method (i.e., the standing and lying time assessed by a 3-dimensional accelerometer).
• High THI only slightly affect the measurement accuracy.

## Graphical Abstract

**Figure:**
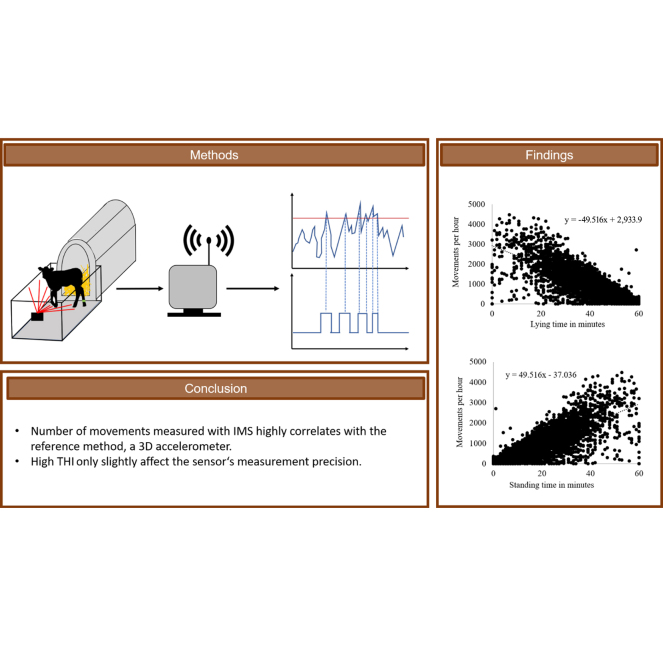
**Summary:** The lying and standing times of preweaning dairy calves (1-7 days of age) were monitored with a 3-dimensional accelerometer and an infrared sensor monitoring system (IMS). The IMS converted a movement-induced change in the heat pattern emitted by the calf's body into an analog signal. These signals were counted and condensed into an absolute number of movements. The Pearson correlation coefficient between both standing and lying time and the number of movements measured by the IMS was r = 0.85 and r = -0.85, respectively. The coefficient of determination (R^2^) varied from 0.47 to 0.93 among calves. Only high temperature-humidity indexes (THI; >72) slightly affected the measurement accuracy, but still showed comparable results.

On today's dairy farms, technologies for monitoring animal behavior in dairy cattle are widely used for various purposes such as detection of estrus, mastitis, or calving. The use of real-time monitoring technologies for farm or animal management has been termed as precision livestock farming (Halachmi and Guarino, 2016). This includes monitoring technologies for farm equipment (e.g., production measurements), and technologies that can be worn by the animal to measure individual data and detect temporal physiological variability. Devices that are worn by the animal are used to assess individual indicators for physiological changes and animal welfare (e.g., movement, feeding, or lying behavior; Costa et al., 2021). The use of sensors has been widely adopted in the dairy industry to monitor dairy cows (Rutten et al., 2013). For calf rearing and heifer management, however, sensor use is lagging behind, although it could provide important information about health and performance. A variety of sensors have already been evaluated in calves. There are sensors that get attached to the feet to assess lying behavior, locomotion, and step activity (de Passillé et al., 2010; Bonk et al., 2013; Swartz et al., 2016) or to the calves' neck or ears to assess rumination behavior (Burfeind et al., 2011; Hill et al., 2017), feed intake, and suckling (Hill et al., 2017; Roland et al., 2018). Sensors attached to an animal, however, have disadvantages such as sensor losses, power issues, stressful manipulation when attaching the sensor, and perception of invasiveness. Recently, a pyroelectric infrared sensor monitoring system (**IMS**; Calf Monitoring System, Futuro Farming GmbH) became commercially available, which can be attached to the fence of a calf hutch avoiding these disadvantages. However, infrared technology can be influenced by the ambient temperature-humidity index (**THI**), potentially limiting the accuracy of the measurement devices (Cantor et al., 2022). This monitoring system has not been validated yet but could help identify sick calves and allow more timely intervention.

Therefore, the objective of the study was to associate the movement assessed with an IMS in preweaning dairy calves with total lying and standing time assessed by a 3D accelerometer at different ambient temperatures. We hypothesized that (1) the data generated by the infrared sensor are highly correlated with an established gold standard reference method (i.e., the lying and standing time obtained by 3-dimensional accelerometer data logger, **3D accelerometer**; Hobo Pendant G Data Logger, Onset Computer Corporation) attached to the right hind leg of the calves, and (2) THI does not influence the measuring accuracy of the IMS.

The study was conducted from July to September 2021 on a commercial dairy farm in Germany (latitude, 52.447417; longitude 14.183622). The study protocol was in accordance with the Institutional Animal Care and Use Committee of the Freie Universität Berlin (approval number: 2340–16–2021). A sample size according to Bonk et al. (2013) and Roland et al. (2018) was used, including 19 and 15 calves, respectively.

A total of 35 preweaning Holstein Friesian dairy calves were enrolled in the study. Calves were 1 to 7 d old and considered healthy by the absence of visible clinical symptoms at the beginning of each trial. Individuals with a total respiratory score >4 or a fecal score ≥2 according to the calf health scoring chart (McGuirk and Peek, 2014) were excluded from the study.

Calves were kept individually in calf hutches (2.05 × 1.15 × 1.35 m) with an adjacent paddock (1.50 × 1.10 m) and bedded on straw. The lying and standing behavior was monitored with a 3D accelerometer (Hobo Pendant G Data Logger, Onset Computer Corporation, Bourne, MA) attached to the right hind leg of the calves. The 3D accelerometer was wrapped with cotton wool (Hartmann cotton 100 g, Paul Hartmann AG, Heidenheim, Germany) and a cohesive elastic wrap bandage (CoFlex Vet, Andover Healthcare, Salisbury, MA) to avoid pressure points. The wrapped data logger was attached to the lateral side of the calf's right hind leg above the metatarseal joint using a textile double-sided Velcro strap (Brand One-Wrap 25 mm, Velcro GmbH, Freiberg am Neckar, Germany). According to Bonk et al. (2013), the recording frequency was set to one frame per minute. Data of the 3D accelerometers were downloaded after the observation period of 14 d using the manufacturer's software (version 3.7.22; Onset Computer Corp.) and exported as one CSV file per calf. A custom-built script for data processing was created using the Python programming language (van Rossum and Drake, 1995) and utilizing the data analysis and statistics library pandas (McKinney, 2011). The output with the aggregated results and processing meta-data were stored as an Excel XLSX file (Office 2010, Microsoft Deutschland Ltd., Munich, Germany). The degree of vertical tilt (y-axis) was used to determine the lying position of the animal. Readings ≥120° and <120° indicated the calf standing and lying, respectively (Bonk et al., 2013). For each calf, PDF files with y-axis line graphs were created for visual inspection to verify and ensure accurate data processing of the script. Data of 1 h (n = 60) were summarized to 1-h periods.

A pyroelectric IMS (Calf Monitoring System, Futuro Farming GmbH, Regensburg, Germany) was fixed to the middle of the paddock fence facing the interior of the calf hutch with a maximum distance of 3.55 m to the calf and an angle of maximum 55°. Maintaining this angle and distance ensured that movements in the hutch were also recorded in the back of the hutch and shadowing was kept to a minimum. The IMS converted a movement-induced change in the heat pattern emitted by the calf's body into an analog signal that is detected as movement when a threshold (25 Hz) is exceeded. These signals were counted and condensed into an absolute number indicating the movements per 5 min. Data were summed up manually to 1-h intervals using MS Excel (Office 2010, Microsoft Deutschland Ltd.). The observation period for each calf lasted 14 consecutive days. We assumed that a lying calf shows little movement, whereas a standing calf will mostly run, jump, or play and will therefore show a higher number of movements (Gladden et al., 2020). Based on this assumption, number of movements per hour detected by the IMS was compared with the lying and standing time assessed with the 3D accelerometer.

Data loggers (Tinytag Plus II, Gemini loggers Ltd., Chichester, UK) to record temperature (**TEMP**) and relative humidity (**RH**) were placed centrally at the inner roof of each calf hutch at a height of 1.35 m and outside on top of the roof of one calf hutch. Another logger was located in a rain- and sun-protected area near the calf hutches (10 to 30 m) at a height of 2 m. The loggers measured TEMP (−25 to 85°C) and RH (0% to 100%) every 10 min. The THI was calculated with the equation described by Mader et al. (2006):

THI = (0.8 × dry bulb TEMP) + [(RH/100) × (dry bulb TEMP − 14.4)] + 46.6.

Furthermore, the averages of TEMP, RH, and THI data were calculated in 1-h intervals using MS Excel (Office 2010, Microsoft Deutschland Ltd.).

In dairy cows, THI between 68 and 72 are considered heat stress and cause a decline in milk production (Ravagnolo et al., 2000; Zimbelman et al., 2009) and reproduction (Schüller et al., 2014). In calves, there are only few studies on THI thresholds. According to Dado-Senn et al. (2020), calves should be monitored closely when THI reaches 65 to 69 to minimize the risk of heat stress.

For this study we defined 3 THI categories (i.e., low THI [<68], medium THI [68–72], and high THI [>72]) to evaluate the accuracy of the IMS at different THI.

Pearson correlation coefficients between the number of movements assessed by the IMS and standing minutes measured by the 3D accelerometer were calculated using MS Excel (Office 2010, Microsoft Deutschland). Linear regressions for individual calves were calculated using SPSS (SPSS Inc., IBM).

Initially, 35 dairy calves were enrolled in the study. Fourteen calves had to be excluded due to loss (n = 10) or malfunctioning of the 3D accelerometer (n = 4). One calf was euthanized due to septicemia during the study. In total, 20 calves aged 3.8 ± 2.1 d were considered in the final statistical analyses. The 14-d observation periods resulted in 329 ± 12.0 one-hour intervals per calf (Table 1). Throughout the entire trial, 13,440 one-hour intervals were assessed by the IMS as the sensor measured data continuously. The number of 1-h intervals of lying and standing time assessed by the 3D accelerometer was 8,184 and 8,182, respectively. After exclusion of 1,603 intervals due to a malfunctioning of the 3D accelerometers, 6,581 one-hour intervals of standing time and 6,579 one-hour intervals of lying time were used for the final analyses. The 1-h intervals assessed by the 3D accelerometer were compared with the corresponding 1-h intervals assessed by the IMS. There were no outliers filtered out for the final analyses.

**Table 1:** The linear regression relationship of lying times and movement for individual calves showing number of measurement intervals, coefficient of determination, intercept, 95% confidence intervals, and slope

Calf no.	N	R^2^	Intercept	95% CI	Slope	95% CI
1	343	0.93	57.61	57.09 to 58.17	−0.014	−0.015 to −0.014
2	344	0.69	56.44	55.62 to 57.25	−0.018	−0.019 to −0.017
3	343	0.47	53.47	51.98 to 54.95	−0.018	−0.020 to −0.016
4	345	0.81	56.66	55.83 to 57.49	−0.014	−0.015 to −0.013
5	345	0.87	56.70	55.90 to 57.50	−0.018	−0.019 to −0.017
6	345	0.74	56.79	55.79 to 57.80	−0.020	−0.021 to −0.019
7	345	0.71	55.75	54.79 to 56.71	−0.018	−0.019 to −0.016
8	311	0.59	53.78	52.51 to 55.05	−0.017	−0.018 to −0.015
9	322	0.81	57.36	56.65 to 58.07	−0.015	−0.016 to −0.014
10	320	0.79	57.14	56.32 to 57.97	−0.015	−0.016 to −0.014
11	324	0.77	55.98	55.32 to 56.65	−0.016	−0.017 to −0.015
12	321	0.83	57.85	57.26 to 58.45	−0.011	−0.012 to −0.011
13	322	0.65	53.49	52.46 to 54.51	−0.015	−0.016 to −0.014
14	322	0.83	56.01	55.27 to 56.75	−0.014	−0.015 to −0.014
15	320	0.83	55.89	54.76 to 57.01	−0.011	−0.012 to −0.010
16	322	0.85	56.46	55.78 to 57.13	−0.014	−0.014 to −0.013
17	321	0.90	57.92	57.38 to 58.46	−0.016	−0.017 to −0.016
18	322	0.84	55.61	54.89 to 56.33	−0.015	−0.016 to −0.015
19	321	0.89	56.69	56.04 to 57.33	−0.014	−0.014 to −0.013
20	321	0.81	55.07	54.23 to 55.90	−0.018	−0.019 to −0.017

The average standing and lying time assessed by the 3D accelerometer was 13.4 ± 12.7 (mean ± SD) min/h and 46.6 (±12.7) min/h, respectively. The median (25th percentile; 75th percentile) number of movements measured by the IMS was 360 (60; 919) movements per hour. The average TEMP was 19.4 (±5.6)°C with a minimum of 6.2 and a maximum of 38.6°C. The average THI was 64.8 (±7.5) with a minimum of 43.1 and a maximum of 85.5.

The Pearson correlation coefficient between both standing and lying time (min) and the number of movements measured by the IMS was r = 0.85 and r = −0.85, respectively (Figure 1). Linear regressions including intercept and slope of the 20 calves are summarized in Table 1. If the 95% confidence intervals from each calf overlap, they do not differ significantly from each other.

**Figure 1:**
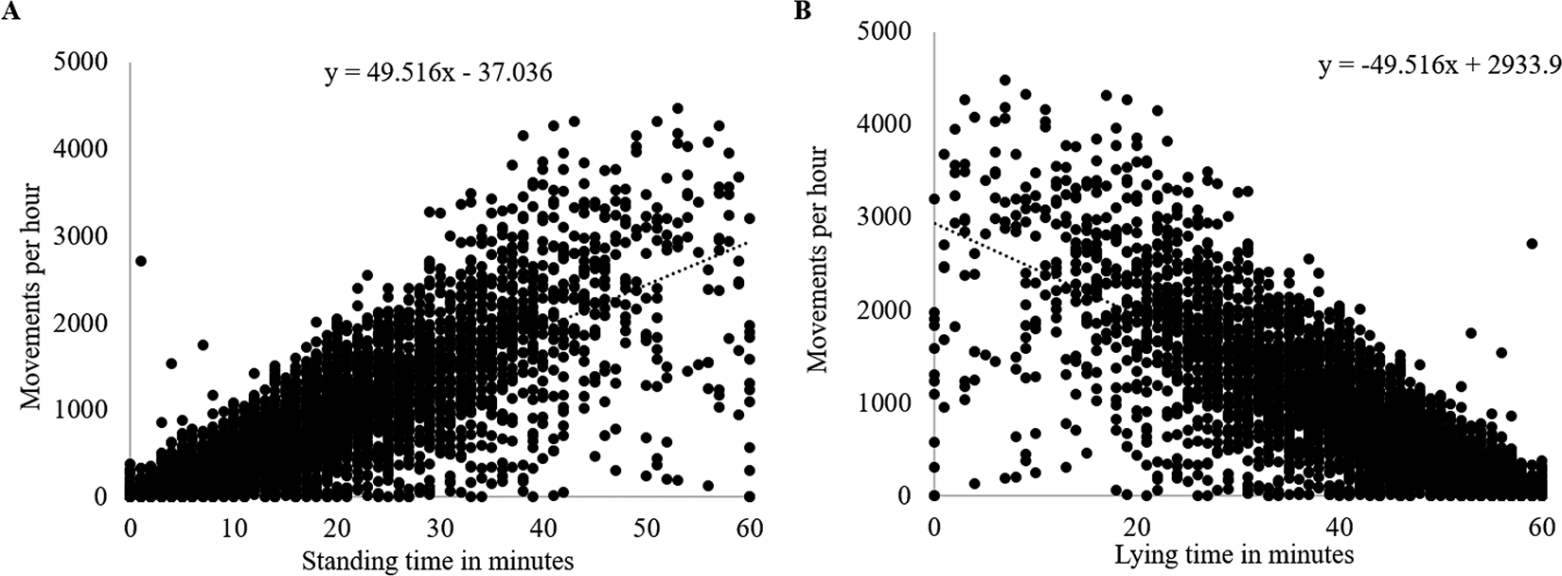
Comparison of total standing time (A) and total lying time (B) in minutes per hour of 20 dairy calves determined by a 3-dimensional accelerometer (Hobo Pendant G Data Logger, Onset Computer Corporation) and number of movements per hour measured with a pyroelectric infrared sensor monitoring system (Calf Monitoring System, Futuro Farming GmbH; A: 6,580 one-hour intervals; R^2^ = 0.7223; r = 0.85; B: 6,581 one-hour intervals; R^2^ = 0.7223; r = −0.85).

In the statistical analysis, 4,601, 948, and 1,032 one-hour intervals of standing time were considered for low, medium, and high THI category, respectively. The Pearson correlation coefficients comparing total standing time and number of movements per hour considering THI category were 0.86 (95% CI: 0.85–0.87; *P* < 0.001), 0.85 (95% CI: 0.82–0.87; *P* < 0.001), and 0.81 (95% CI: 0.78–0.84; *P* < 0 < 0.001) for low (<68), middle (68–72), and high (>72) THI, respectively (Figure 2).

**Figure 2:**
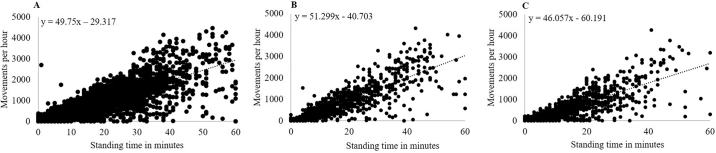
Comparison of total standing time in minutes per hour of 20 dairy calves and amount of movements per hour considering temperature-humidity index (THI). (A) At low THI (>72) the Pearson correlation coefficient was r = 0.86 (R^2^ = 0.7361; n = 4,601). (B) At medium THI (68–72) the Pearson correlation coefficient was r = 0.85 (R^2^ = 0.7204; n = 948). (C) At high THI (>72) the Pearson correlation coefficient was r = 0.81 (R^2^ = 0.6546; n = 1,032).

The objective of the study was to evaluate if an IMS is suitable to detect movement in preweaning dairy calves from a distance. Our data show that the number of movements measured by the IMS were highly correlated with the chosen gold standard reference method (i.e., the standing and lying assessed by a 3D accelerometer).

Several parameters can be estimated by 3D accelerometers (e.g., lying and standing time, lying bouts, and number of steps; Belaid et al., 2020). In this study, we focused on lying and standing time. The average lying time detected in the present study (46.6 ± 12.7 min/h) was similar to data previously described (37.72 ± 24 min/h: Swartz et al., 2016; 44.9 ± 21.3 min and 42.5 ± 13.4 min: Belaid et al., 2020). Newborn calves spend most of their time resting. With increasing age, lying time decreases (Hänninen et al., 2003).

Our chosen gold standard reference method, the Hobo Pendant G Data Logger, has been already validated by plotting the 3D accelerometer data against direct or video observations (Bonk et al., 2013; Swartz et al., 2016). The Pearson correlation coefficients between the 3D accelerometer and the IMS were high (r = 0.85), which confirms our hypothesis that the IMS is suitable to detect movement in dairy calves. Compared with the 3D accelerometer, however, the IMS is not able to measure total lying and standing times, but can detect changes in the position of the calf.

Pyroelectric infrared sensors have already been evaluated to detect movement in humans (Hao et al., 2006). They can even be used to classify the direction of movement, the distance of the object from the sensor, and the speed of movement with an accuracy of 92% (Yun and Lee, 2014). In calves, infrared technology has only been used to measure body temperature (Scoley et al., 2019; Cantor et al., 2022) or respiration rate (Lowe et al., 2019). To our knowledge, this technology has not yet been used to detect movement in preweaning dairy calves. The coefficient of determination (R^2^), however, varied from 0.47 to 0.93 among calves. The color pattern and hair texture of adult cows has been demonstrated to affect the heat emission (Isola et al., 2020) but was not considered in our study, which could be a reason for the high range of R^2^ within the individual calves. Other possible factors affecting variability could be manipulation of the sensor, higher activity or inactivity of the calves, or higher THI due to solar exposure of the calf hutches. Further research is warranted to study if and how these factors influence the accuracy. Influence of THI category affected coefficients of correlation only slightly (low THI: 0.86; medium THI: 0.85; high THI: 0.81). The confidence intervals of the different THI categories overlapped, besides a marginal lower confidence interval at high THI, indicating that low and medium THI do not affect the accuracy of the IMS. Only high THI (>72) slightly affected the measurement accuracy, but still showed comparable results.

The effect of heat stress in calves has not been studied to the same extent as in in dairy cows. Nevertheless, heat stress in calves can change behavior and cause acute stress (Bakony and Jurkovic, 2020). Kovács et al. (2018) observed changes in lying bout frequency and total lying time in heat-stressed calves, but so far, research on number of movements does not exist yet.

The fact that the IMS are attached to the fence or other structures minimizes the risk of loss and invasiveness to the animal. At the same time, however, the attachment within reach also involves a risk of interference between the calf and the sensor system, potentially influencing the assessment of data.

A limitation of our study was the high number of individuals excluded due to a loss of the 3D accelerometer, especially in the beginning of the trial. The reason for the high loss rates could be the type of Velcro leg band that was used to attach the 3D logger in the beginning of the study. After replacing the model of Velcro bandage, no further losses were observed. In a comparable study (Bonk et al., 2013), Vet wrap bandage was used to attach the sensor at the calves' leg and there was no mention of loss. In addition to the risk of loss, major disadvantages of animal-attached sensors are the limits on memory, power, and the potential influence on the animal's behavior by being potentially invasive (Rushen et al., 2012). The IMS could be an alternative to avoid such limitations, and furthermore, allows real-time monitoring. So far, the IMS can only be used for single-housed calves. Most diseases, however, occur in the first weeks of life (Urie et al., 2018), and often calves in that age bracket are kept individually. Increasingly, group housing is preferred to individual housing, due to its positive effects on social skills, solid feed intake preweaning, and weight gains before and after weaning (Costa et al., 2016).

A limitation of the study was the lack of an a priori sample size calculation. We used a sample size according to Bonk et al. (2013) and Roland et al. (2018), including 19 calves with an observation period of 24 h and 15 calves with an observation period of 60 h, respectively.

Another limitation is the comparison of lying and standing time with movements. Standing times and movements often coincide (Gladden et al., 2020), but calves standing idle (Pempek et al., 2017) might not be assessed by the IMS.

Our data show that the number of movements of dairy calves measured by an IMS were highly correlated with the gold standard reference method (i.e., the standing and lying time assessed by 3D accelerometer). Only high THI (>72) slightly affected the measurement accuracy of the IMS. For a future validation, video data should be generated to avoid possible confounding such as direct solar exposure, manipulation of the sensor by the calf or calf activity, and ability to correlate comparable data. In our study the IMS was only applied to Holstein Friesian calves in their first 1 to 7 d of life. Therefore, further research is warranted to evaluate this device for older calves and the potential for recognizing movement patterns indicative of disease or stress.
